# Heritability and Genetic Correlation of Age at First Egg and Egg Number up to 40 Weeks of Age After Long-Term Selection in Taiwan Indigenous Chicken

**DOI:** 10.3390/ani15111534

**Published:** 2025-05-23

**Authors:** Der-Yuh Lin, Chia-Te Chu, Mu-Yao Lin, Ming-Yang Tsai, Shwu-Jen Tzeng, Ming-Che Wu, Hsiu-Luan Chang

**Affiliations:** 1 Genetics and Physiology Division, Taiwan Livestock Research Institute, Ministry of Agriculture, Tainan 71246, Taiwan; linderyuh@gmail.com (D.-Y.L.); joeychu18@tlri.gov.tw (C.-T.C.); mcwu71246@gmail.com (M.-C.W.); 2 Department of Biotechnology and Bioindustry Sciences, National Cheng Kung University, Tainan 70154, Taiwan; 3 Formosan Farmers Association for Swine Improvement, Taipei 24242, Taiwan; ellk394@gmail.com; 4 Livestock Management Division, Taiwan Livestock Research Institute, Ministry of Agriculture, Tainan 71246, Taiwan; mytsai@mail.tlri.gov.tw; 5 Department of Medical Laboratory Science and Biotechnology, Chung Hwa University of Medical Technology, Tainan 71703, Taiwan; shwujen20200626@gmail.com; 6 Department of Animal Science, National Pingtung University of Science and Technology, Pingtung 91201, Taiwan

**Keywords:** animal model, chicken, genetic parameter, laying trait, selection

## Abstract

The objective of this study was to estimate the genetic parameters of the egg-laying traits of hens after long-term selection. Four lines were derived from Taiwan indigenous chickens conserved in situ by the National Livestock Biodiversity Program. A total of 9834 hens’ laying records from 18 non-overlapping generations classified as seven generational stages from 2006 to 2023 was used. The traits evaluated included body weight at 16 weeks of age, age at the first egg (Sday), body weight change between Sday and 40 weeks of age (Gain40), mean egg weight at 40 weeks of age (EW40), and egg number up to 40 weeks of age (EN40). A four-trait animal model via the REML procedure was used to estimate the variance/covariance components for each generational stage within each line. The ranges of the heritability estimates for Sday, Gain40, EW40, and EN40 were 0.09–0.61, 0.03–0.70, 0.32–0.83, and 0.00–0.65, respectively. Within the lines’ generational stages, most of the heritability estimates of Gain40 and EW40 were higher than those of Sday and EN40. Also, less fluctuation was found in the heritability estimates of Gain40 and EW40 among the generational stages within the lines. Favorable negative genetic and phenotypic correlations between Sday and EN40 were observed at the last generational stage, which could be beneficial for future breeding programs to improve egg production.

## Simple Summary

We estimated the genetic parameters of egg-laying-related traits in different generational stages of four Taiwan indigenous chicken lines. All four lines were derived from in situ conservation populations of the National Livestock Biodiversity Program and had undergone long-term selection for egg-laying traits since 1987. The results showed that the estimated genetic parameters of the investigated traits fluctuated among the generational stages, and the extent of fluctuation varied by line. The heritability and correlation estimates of the traits of the final stage of the selected generations suggest that the sustainability of breeding programs and the selection of the four traits from this study can still be improved.

## 1. Introduction

In Taiwan, broilers are divided into two types: white broilers and colored broilers. Colored broilers were important as a large share of Taiwan’s poultry industry and have diverse varieties. Among them, ‘red-feathered’ and ‘black-feathered’ broilers are the main ones, accounting for more than 85% of the colored broiler market. However, there are still native chickens in Taiwan, and their phenotypic characteristics vary from region to region. They are commonly known as Taiwan indigenous chickens. Although the egg production, growth performance, and other productivity values of Taiwan indigenous chickens are lower than those of the above-mentioned broilers and their genetic resources are of near-threatened status, their premium meat quality makes their selling price much higher than other broilers in Taiwan. Therefore, maintaining excellent meat quality and improving egg production capacity are the keys to the sustainable development of Taiwan’s indigenous chicken industry.

The National Livestock Biodiversity Program (NLBP) was set up in 1987 as part of an in situ conservation plan for Taiwan’s indigenous livestock species. In situ conservation populations of Taiwan indigenous chickens were thus established based on local farms, which were the founder populations of the experimental lines used in this study. The four experimental lines, L7, L9, L11, and L12, originated from farms at Taichung, Chiayi, Tainan, and Taitung counties in central, South/central, Southern, and Eastern Taiwan, respectively [1,2,3].

Egg production efficiency is an important economic indicator in the chicken industry and can be assessed by various laying traits, including age and body weight at onset of egg production, egg number, egg weight, etc. Studies have indicated that the onset of egg laying is a signal of sexual maturity in hens, which is regulated by genetic factors [4,5]. The trait recorded in these studies was the age at first egg, which was related to body weight [6,7]. The reason for this was that a specific threshold weight was necessary to initiate and maintain egg production in egg-type and meat-type chickens, such as in Japanese quail [8]. Moreover, body weight at first egg and throughout the production year influenced egg production efficiency. The literature has shown that the body weight, egg weight, egg number, and egg mass of hens could be increased with age up to one year [9], but the egg number is relatively associated with the age at first egg and an egg production peak at about 30 weeks is reported, which varies among flocks due to genetic factors [6,7].

Genetic parameter accuracy is essential for breeding value prediction and thus selection in breeding programs [10,11,12]. However, one can change the genetic variances after selection, with the changed magnitude varying by the selection intensity and the initial allele frequencies [11,12]. The literature has indicated that additive genetic variance, and thus the heritability of chicken, has changed over a long term breeding program, similarly to other farm animals under selection [13]. Therefore, it is essential to update genetic parameters regularly for faster generation turnover, thus increasing genetic progress. The purpose of this study was to use a multi-trait animal model to estimate the heritability and genetic correlations of Taiwan indigenous chickens under long-term directional selection and, thus, to understand the feasibility of continued improvements in economically important laying-related traits.

## 2. Materials and Methods

### 2.1. Inbreeding Coefficient Estimation

This study was conducted from 2006 to 2023 at the experimental chicken farm of Taiwan Livestock Research Institute, and dataset was stored in the Animal Genetic Resource Information Network (ANGRIN).

A total of 9834 hens’ laying records from 2006 to 2023 over 18 (non-overlapping) generations, from G1 to G18, were analyzed. All G1 animals have known parent and untraceable grandparent lineages; thus, G1 is considered as basal generation. Chicks were weighted and tagged with identification tag on hen wing right after hatching and then raised on a litter floor with 24 h of light per day in the first week. From the second week of age, the light was gradually shortened to 16 h per day until five weeks of age. From six to eighteen weeks of age, natural light was provided. Then, the chickens were transferred to individual battery cages with restricted feeding. During the egg-laying period, there was a total of 13 h of basic light at night, which was supplemented by artificial light. Thereafter, the chicken’s light exposure was increased by 15 min per week until their total light exposure was 17 h per day. All experimental chickens were maintained under the same housing conditions and fed with the same diet.

Two traits for selection were as follows: (1) selection for the desired morphological characteristics based on body conformation at 10 weeks old and then body weight at 16 weeks of age (W16), with top 50% of females and top of 10% of males being selected for each generation; and (2) selection for egg number from the first egg laid to 40 weeks of age (EN40), with the top 30% of hens being selected for producing fertilized eggs later. Hens over 40 weeks of age were artificially inseminated with roosters that had no common ancestors within three generations to produce the breeding candidates for next generation. This was expected to slow the inbreeding rate, thereby avoiding a drastic decline in performance, particularly in egg-laying traits. Each selected rooster was mated with 5–7 hens by artificial insemination within line, and the fertilized eggs were hatched in batches. Traits in the study included body weight at l6 weeks of age (W16), age at first egg (Sday), mean egg weight at 40 weeks of age (EW40), egg number up to 40 weeks of age (EN40), and body weight changes between Sday and 40 weeks of age (Gain40).

### 2.2. Heritability and Correlation Estimation

The accuracy of genetic parameter estimation is sensitive to sample size (number of individuals with phenotype) and often requires large datasets. Due to the limited number of laying hens in each generation within lines, the 18 non-overlapping generations were further divided into seven generational stages, GS1 to GS7. Generational stages started with the base generation (G1) as GS1, then GS2 to GS6, with three generations in each stage. The last stage, GS7, included the last two generations, G17 and G18. The pedigree used in animal model to estimate genetic parameters of each category within lines was traced back to the basal generation (G1). In the 18-generation full pedigree list, inbred animals of the L7, L9, L11, and L12 accounted for 82.2%, 81.7%, 77.2%, and 80.2% respectively. A summary of laying hens’ lineage by line is shown in Table 1. The laying hens for each line in this study were derived from 182 to 204 sires and 651 to 826 dams, with the dam/sire ratio varying between 3.57 and 4.05 depending on the line (Table 1). Moreover, ratios of paternal grand-dams to paternal grand-sires and maternal grand-dams to maternal grand-sires within lines ranged from 1.28 to 1.43 and from 1.84 to 2.15, respectively. On average, maternal grand-sires had twice as many as paternal grand-sires, and maternal grand-dams had three times as many as paternal grand-dams.

Statistical analysis was at the hen level. Descriptive statistics of the Sday, Gain40, EW40, and EN40 were obtained using the PROC UNIVARIATE procedure in SAS release 9.4 [14]. The model used to estimate the genetic parameters of each generational stage from GS1 to GS7 within lines was multivariate four-trait (Sday, Gain40, EW40, and EN40) animal model. Variance and covariance components were estimated by derivative-free restricted maximum likelihood (DFREML) procedure using the statistical software (VCE 6.0) described by Groeneveld et al. [15], and thus, phenotypic variances, heritability, and correlations were determined. Therefore, the four-trait linear model was formed as follows:

*y* = *Xβ* + *Zd* + *e*

where *y*, *β*, *d*, and *e* are vectors of the observations of traits, fixed effects (including year/month classes of hens and their sires hatched as classification factors and W16 as covariate), random direct genetic effects, and random residuals, respectively; *X* and *Z* are incidence matrices relating vectors *β* and *d* with *y*. Random effects are distributed as centered normal distributions with variance–covariance matrices equal to the following:

$$V\left[\begin{array}{c} d\\ e \end{array}\right]=\left[\begin{array}{cc} G⊗A & 0\\ 0 & R⊗I \end{array}\right]$$

where ⊗ is the Kronecker product operator, *A* is the additive genetic relationship matrix (pedigree traced back to G1), and *G* and *R* represent the (co)variance matrices between traits for direct genetic and residual effects, respectively. *I* is identity matrix of appropriate size.

The variance components for direct genetic effects (σ^2^_d_) and residuals (σ^2^_e_) were used to calculate the phenotypic variance (σ^2^_y_) and thus heritability (h^2^) as σ^2^_y_ = σ^2^_d_ + σ^2^_e_ and h^2^ = σ^2^_d_/σ^2^_y_. Moreover, the genetic (*r_g_*) and phenotypic (*r_p_*) correlations between the ith and jth traits were estimated as *r*_g_ $=Cov\left(d_{i},d_{j}\right)/\left(\sigma_{d_{i}}*\sigma_{d_{j}}\right)$ and $r_{P}=Cov\left(y_{i},y_{j}\right)/\left(\sigma_{y_{i}}*\sigma_{y_{j}}\right)$.

## 3. Results

### 3.1. Laying Traits of Chicken Lines

The descriptive statistics of their traits are summarized in Table 2. The L7 and L12 hens had a heavier average body weight at 16 weeks of age (W16) than the L9 and L11 hens, but they laid their first eggs later (Sday). The range of Sday for the lines investigated was 116–222 days of age with a CV of 7.9–10.2%. Gain40 varied widely from an 870 gm weight loss to a 1897 gm weight gain with a CV of 74.8–113.8%. Most hens gained weight between the time they first started laying eggs and 40 weeks of age, but 7%, 10%, 9%, and 17% of the L7, L9, L11, and L12 hens, respectively, lost weight. The 95% confidence interval for EW40 was 37.6–54.2 gm, which was classified as the peewee to medium egg grade based on USDA egg size standards. Although the time from Sday to 40 weeks of age varied among the lines, the average was about 130 to 135 days. This suggested some hens laid their first egg early, e.g., at 116 days of age. Therefore, the maximum EN40 of the L7, L9, L11, and L12 hens reached 133, 143, 145, and 132 eggs, respectively. Moreover, the mean EN40 of the L12 hens was lower than the other three lines, with an average value of 77.1 eggs and a 95% confidence interval of 34 to 106 eggs. When the uniformity of the traits was evaluated according to the CV, it was found that Gain40 had the largest variation (>70%), followed by EN40 (>20%) and W16 (>15%). EW40 and Sday had less variation, with a CV range of about 8–10%.

The differences among the phenotypic means of the lines for the traits in Table 2 were tested using an F-test, and all traits considered were significant at *p* < 0.0001. Furthermore, a *t*-test was conducted for a pairwise comparison between lines. L7 and L12 had heavier W16 values than L11 and L9 on average (*p* < 0.001), and L11 showed a heavier W16 value than L9 (*p* < 0.0001). L9 and L11 laid their first eggs at a similar age (*p* = 0.2237), but both lines laid their first eggs earlier than L7 and L12 (*p* < 0.0001). Also, Gain40, EW40, and EN40 showed significant differences in all the pairwise comparisons between the lines at *p* < 0.0001, <0.0001, and <0.05, respectively.

According to the arguments presented by Byrne [16] and Hair et al. [17], the data were considered normal if the skewness was between −2 and +2 and the kurtosis was between −7 and +7. As shown in Table 2, except for the Sday value of L11, the skewness values of the traits studied ranged from −0.95 to 0.75, indicating that the data distribution was close to symmetrical. Likewise, kurtosis values ranged from −0.66 to 2.60, except for the Sday value of L11. Therefore, the traits discussed in this study were considered as normal univariate distributions.

Dividing the 18 non-overlapping generations (G1 to G18) into seven generational stages, GS1 to GS7, the inbreeding coefficients (F) of the generational stages within the lines are summarized in Table 3. Due to the assumption of a non-inbred base generation (G1), the inbreeding coefficient of GS1 was set to zero: F = 0. Before the fifth generational stage (GS5), the average inbreeding level within the lines was below 0.25. In addition, the average inbreeding coefficient of L12 in each generational stage was higher than that of other lines. Also, the mean inbreeding coefficients among the lines within the generational stages were significantly different (*p* < 0.05).

We checked the full pedigree for each studied line and found that there were two full-sib hens of L7 with high inbreeding coefficients (=0.5156 > 0.5). They were produced by a pair of full-sibs, which also descended from full-sib mating; i.e., the grandparents were full-sibs too. Furthermore, the great-grandparents were paternal half-sibs. However, we re-ran the inbreeding coefficient for all animals in the pedigree using MATVEC [18] and the INBREED procedure from the SAS package [14]. The results showed that the inbreeding coefficients obtained from VCE6.0, MATVEC, and SAS were 0.5156, 0.5154, and 0.5154, respectively.

### 3.2. Effect of Inbreeding on Body Weight at 16 Weeks of Age

**Figure 1:**
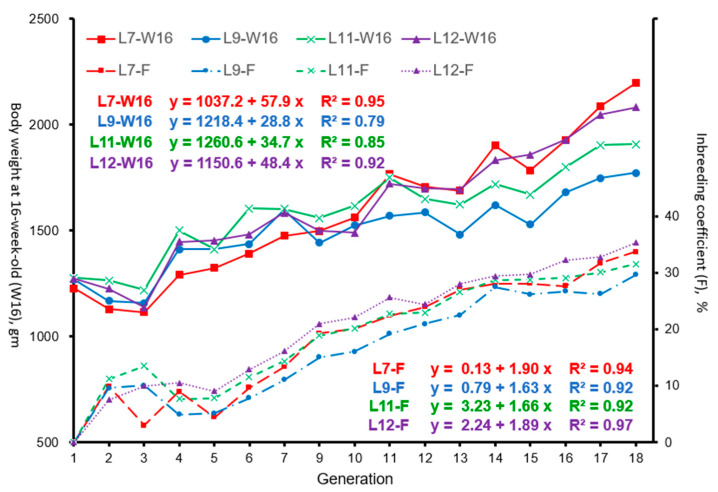
Body weight of hens at 16 weeks old (W16) and inbreeding coefficient (F) according to generation number in Taiwan indigenous chicken lines.

The trends of body weight at 16 weeks of age (W16) and inbreeding coefficient (F, %) of the laying hens in the generation number by line are presented in Figure 1.

Comparing the phenotypic improvements of W16 between the first generation (G1) and the last generation (G18), weight gain was 970, 504, 630, and 809 gm in L7, L9, L11, and L12, which accounted for 79, 40, 49, and 64% increments, respectively. The linear regression coefficient of W16 according to the generation number showed that the phenotypic progress per generation was larger in L7 and L12 (57.9 and 48.4 gm, respectively) than in L9 and L11 (28.8 gm and 34.7 gm, respectively). L7 and L12 showed, on average, greater inbreeding than L9 and L11 in the last two (17th and 18th) generations. In the third generation (G3), W16 showed a significant selection impression in all lines evaluated. F (%) continued to increase with the generation number starting from the fifth generation (G5), but W16 did not show any adverse effects of inbreeding depression. Similar results were also obtained when the linear regression of the inbreeding coefficient on the generation number was considered. The inbreeding rates of L7 and L12 per generation were 1.90 and 1.89%, respectively, while the inbreeding rates of L9 and L11 were 1.63 and 1.66%.

**Figure 2:**
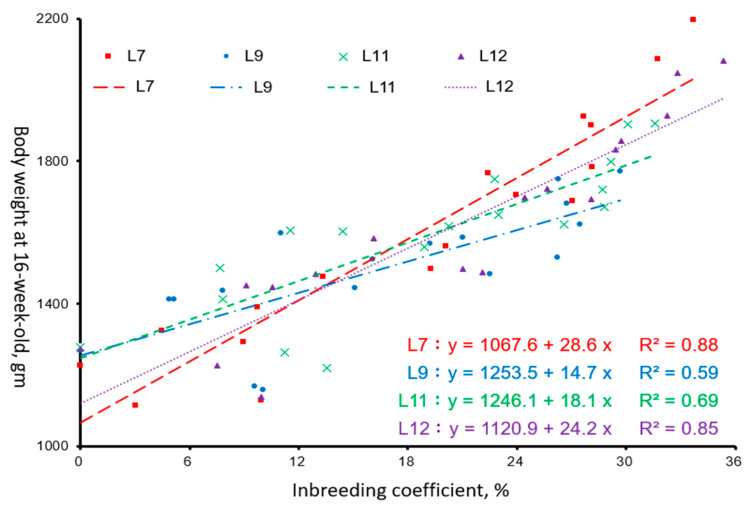
Linear regression of body weight of hen at 16 weeks of age (W16) with inbreeding coefficient in Taiwan indigenous chicken lines.

The linear regression of W16 on its inbreeding coefficient within the lines is presented in Figure 2. The inbreeding coefficients of the last generation (G18) were 33.7, 29.7, 31.6, and 35.3% in L7, L9, L11, and L12, respectively. The average values of W16 in the first generation (G1) were 1227, 1269, 1277, and 1273 gm in L7, L9, L11, and L12 respectively. In our study, within the range of generation-average inbreeding coefficients of 0–36%, each 1% increase in the inbreeding rate (ΔF) resulted in an increase of 14.7 to 28.6 gm in W16, which varied among the lines.

### 3.3. Estimates of Heritability and Correlation

**Figure 3:**
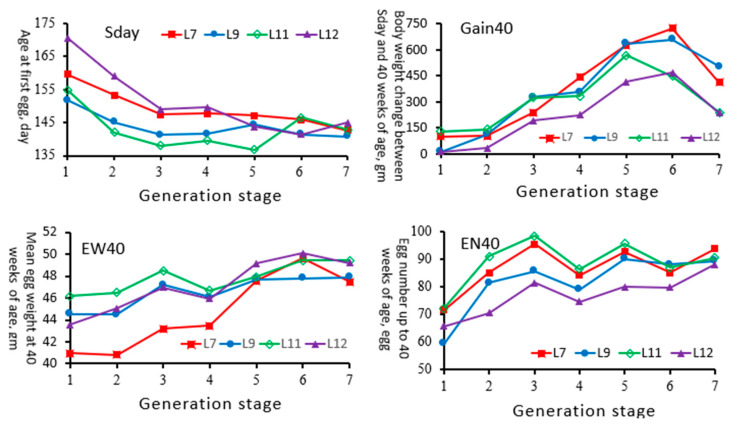
Phenotypic trends of age at first egg (Sday), body weight change between Sday and 40 weeks of age (Gain40), mean egg weight at 40 weeks of age (EW40), and egg number up to 40 weeks of age (EN40) after long-term selection by generational stage in Taiwan indigenous chicken lines.

An overview of the phenotypic trends for the traits evaluated (Sday, Gain40, EW40, and EN40) by generational stage and line is presented in Figure 3. After long-term selection, all traits had developed in a favorable direction, but there were differences among the lines. The Sday value of L12 was decreased from 170 days to 145 days of age along with the EN40 increment from 65 to 89 eggs. The EW40 value of the four lines reached 47–50 g at GS7, but the average weight gain of Gain40 showed a downward trend compared with GS6. This suggested that the variation within lines, including both genetic and non-genetic components, fluctuated over time, and thus, the correlations between traits were unpredictable. Therefore, parameter estimation for each generational stage by line is essential for breeding programs.

Heritability estimates and standard errors for the traits evaluated by generational stage (GS1 to GS7) and line (L7, L9, L11, and L12) are presented in Table 4. One can divide the estimate by the corresponding standard error and refer to the N(0, 1) distribution to test for a significant difference from zero, as shown in Table 4. The heritability estimates of EN40 in the starting generation (G1 = GS1) of each line showed a moderate to high degree of inheritance, with estimates ranging from 0.35 to 0.65. However, the heritability estimates of EN40 in the last generational stage, GS7, were not significantly different from zero at the 5% level (*p* > 0.05), except for L11. This suggested that EN40 might be affected by EW40 and Gain40.

Most estimates for Sday showed values significantly (*p* < 0.05) different from zero and moderate to high-heritability characteristics, ranging from 0.20 to 0.61. Furthermore, less fluctuation in the estimates of Sday among generational stages was observed in L11, ranging from 0.22 to 0.38. It was noteworthy that, with the exception of L11, heritability estimates of Sday showed a decreasing trend when comparing estimates of GS1 with GS7. A similar decreasing trend was also observed in EN40 with the exception of L11.

The Gain40 values of the L7, L9, and L11 laying hens generally showed moderate to high heritability, and all estimates were significantly different from zero, with ranges 0.34–0.51, 0.23–0.70, and 0.17–0.57, respectively. However, low and non-significant heritability estimates of Gain40 were found in GS2 (0.15 ± 0.08) and GS4 (0.03 ± 0.05) of L12. In addition, the heritability estimates of Gain40 were at a moderate level (0.35 and 0.38) in the initial generational stage (GS1) of L7 and L9, while the estimates of GS7 increased to 0.51 and 0.60, respectively, with the increase in generational stage. However, this trend was not shown in L11 and L12. While fluctuations were also observed during GS2 and GS6, the estimates of Gain40 at GS1 and GS7 were more consistent, with estimates of 0.31 and 0.30 for L11 and 0.34 and 0.34 for L12, respectively. The heritability estimate of EW40 for each generational stage of the lines studied was significantly different from zero (*p* < 0.05). When comparisons were made within generational stages in L11 and L12, EW40 had a higher heritability estimate than others, such as Sday, Gain40, and EN40.

Genetic and phenotypic correlations between traits for the initial and last generational stages by lines are presented in Table 5. In the initial generation of the selection, Sday was negatively correlated with others, such as Gain40, EW40, and EN40, both genetically and phenotypically. However, after long-term selection, the genetic and phenotypic correlations between the traits varied in direction and magnitude among the lines. Sday and EN40 showed favorably negative genetic and phenotypic correlations at the last generational stage of each line. Gain40 showed positive genetic correlations with EN40 in L7 and L11, but a negative genetic relationship was observed in L9 and L12. In addition, negative phenotypic correlations of the corresponding traits, i.e., Gain40 and EN40, were also found in all the lines studied. Although EW40 and EN40 had positive genetic and phenotypic correlations at the initial generational stage in L11 and L12, this favorable relationship was found only at the last generational stage of L12 with *r_g_* = 0.42 and *r_p_* = 0.18.

## 4. Discussion

### 4.1. Age at First Egg

The range of the mean Sday for experimental lines (L7, L9, L11, and L12) was 143.4–151.1 days of age, which was earlier than that of Thai native synthetic chickens [19] and those reported by Chinese groups, including Dongxiang and Suken chickens [20], White Leghorn [21], and Beijing-You [21,22], but later than that of Chinese Ningdu Sanhuang [23] hens. Although the variation in Sday can be modified by environmental factors such as lighting, it is determined by genetic background. Furthermore, the literature has shown that the breeds with high egg numbers laid their first eggs earlier than the breeds with fewer eggs due to the genetically negative correlation, as shown in several breeds, such as White Leghorn, Rhode Island Red, Plymouth Rock, synthetic dwarf line, and Beijing-You chickens [24,25]. This was consistent with our results; i.e., on average, the L7, L9, and L11 hens laid their first eggs earlier (before 150 days of age) and had more eggs up to 40 weeks of age (more than 80 eggs) compared to L12.

### 4.2. Effect of Inbreeding on W16

The Pearson correlation coefficient between W16 and the inbreeding coefficient was also significantly positive (r = 0.77 − 0.94 (*p* < 0.001)) in the four chicken lines. Although the selection of morphological characteristics and W16 was the first step in our selection procedure, W16 was not adversely affected by the increase in inbreeding coefficient as the generation number increased, which may be related to the slightly unbalanced selection caused by the mating ratio of non-overlapping generations in this study.

In this study, the mating ratios of dam to sire in the generational stages were from 2.1 to 6.3 with the overall average being 3.8, which deviated slightly from a balanced selection (equal contributions from most families), but not unrestricted selection. Moreover, the results from the simulation study showed that a slightly unbalanced selection had a favorable relation between the retrieved response and loss of genetic diversity compared to a balanced selection at low and moderate heritability levels of small families and populations [26]. A possible explanation could be the slightly unbalanced selection due to differences in family size, which could be more beneficial in terms of the selection response gained and genetic diversity lost [27].

Inbreeding is inevitable, even with random mating, in small and isolated populations, as all individuals are ultimately related to each other. Theoretically, the inbreeding rate (ΔF) is inversely proportional to twice the effective population size: 1/(2Ne). Therefore, based on the average inbreeding rate per generation, the point estimates of Ne in L7, L9, L11, and L12 were 25.2, 28.6, 26.9, and 24.1, respectively, which were smaller than those reported for conservation flocks and field populations of South African indigenous chickens (point estimate ranges of 38.6–78.6 and 118.9–580.0, respectively) [28]. In this study, the inbreeding coefficient increased with generation; meanwhile, the selection response of W16 also manifested with the selection generations. This may be related to the fact that our selection and captive breeding purged the genetic load from the population and that this purging involved part of the lethal or detrimental effects of inbreeding depression; thus, some genetic variation was retained for the selection response expressed. Therefore, from the relationship between the coefficient of inbreeding and W16 in this study, the conclusion was justified that inbreeding per se did not necessarily decrease body weight and that W16 could be increased with selection, which was consistent with the results obtained from albino rats [29] and inbred chickens [30,31]. Although W16 did not show adverse effects as the rate of inbreeding increased, the selection response varied with the lines; L7 and L12 had more of a response than L9 and L11 in terms of the increment of the inbreeding coefficient unit. A possible explanation is that after the consistently directional selection for W16, the general additive effects of desirable genes relating to W16 in L7 and L12 were more likely to express favorable inherent factors than those in L9 and L11 [31,32].

### 4.3. Heritability and Genetic Correlation After Long-Term Selection

When a comparison was made within the generational stages in each line, most heritability estimates of Gain40 and EW40 tended to be higher than those of Sday and EN40, which was consistent with findings by others and confirmed that the heritability of production-related traits was generally higher than that of reproductive traits [7,13,19,33]. In general, genetic parameters can be changed over time after selection [13], and additive genetic variance is one of the critical parameters. Moreover, the changed magnitude depends on the selection intensity and the initial allele frequencies of the selected flocks [12]. Genetic variance and heritability may be due to several potential factors such as genetic drift, the accumulation of inbreeding, and negative covariance between pairs of loci, thus creating a negative linkage disequilibrium under directional or stabilizing selection, which is also called the Bulmer effect [34].

The heritability estimates of the traits investigated in this study were all significant in the initial generational stage and showed moderately to highly heritability. Furthermore, the significantly moderate to high-heritability estimates of Gain40 and EW40 in the last generational stage suggest that the additive genetic variation fluctuated and thus the heritability changed over time after the long-term selection, though continued improvements are still expected. This was also indirectly confirmed the conclusions made by Hill in 2016 [35]. Although Sday and EN40 of GS7 in L11 showed significantly moderate to high-heritability characteristics, this was not the case in L7, L9, and L12, and the corresponding heritability estimates were not significant (*p* > 0.05), with the exception of Sday in L12. The reason for this might be that Gain40 and EW40 are associated with growth traits, while Sday and EN40 are egg-laying traits. In general, it is not easy to maintain sustainable improvements in low-heritability traits, such as Sday and EN40, through long-term traditional selection schemes. However, when traits with low heritability are of interest, selection based on gene ontology principles may be more effective [36,37,38].

Sday and EN40 are related to the development time and fertility of poultry, whereas Gain40 and EW40 are more related to growth rate and body size [39]. The favorable additive genetic correlations between Sday and EN40 found in this study will be beneficial for the Taiwan indigenous chicken production system in selecting for early sexual maturity and improving egg production in hens. This result was consistent with earlier studies for local and commercial chickens, including Thai native synthetic chickens, indigenous Beijing-You chickens, Iranian native chickens, and Mazandaran native chickens [19,22,40,41,42].

In terms of phenotypic and genetic correlations for four traits, the correlation coefficients for each line fluctuated among generational stages, with estimates ranging from negative to positive. Gain40 is defined as the difference between the body weights at 40 weeks of age and the body weight at first egg laid. The body weight of chickens is sensitive to age. Similarly, EW40 refers to the average weight of eggs produced at 40 weeks of age, which involves not only the individual egg weight but also the number of eggs laid at 40 weeks of age as well as the laying hen’s weight.

When the correlation in the last generational stage, GS7, was considered, all experimental lines in this study showed favorable negative genetic and phenotypic correlations between Sday and EN40. Although growth traits are usually negatively correlated with egg production traits or there is no correlation between the two traits [41,43,44,45], this study found that Gain40 and EN40 were positively correlated in L7 and L11, as reported in Horro chickens of Ethiopia [46], and negatively correlated in L9 and L12. When the corresponding phenotypic correlations were considered, Gain40 and EN40 showed a weak negative correlation, which supported the comparison results between different body weight groups of Lohmann white laying hens [47]. One possible explanation is the presence of pleiotropic genes associated with the growth rate and egg production during the laying period. For example, the growth hormone gene is associated with egg production in addition to weight gain [48,49,50].

In summary, estimates of genetic parameters varied considerably by flocks/breeds, which indicated that there were large differences in the genetic basis for different lines, and thus, the estimates from this study presented various selection response over 18 generations. Moreover, the range of phenotypic correlation estimates among the generational stages within the lines is smaller than that of the corresponding genetic correlation as expected, which is due to environmental influences. Therefore, it is important to estimate the genetic correlation before initiating a selection scheme to unveil correlations that might be masked by non-genetic factors, such as the environment.

## 5. Conclusions

Monitoring genetic variation is a promising strategy, not only to ensure the continuation of genetic progress in a selection program but also to provide an understanding of the genetic diversity of conserved indigenous populations. In this study, although the relationship between genetic and phenotype fluctuated with generation stage, it did not show too harmful an effect after long-term selection. Meanwhile, except for EN40 in L9 and L12, the heritability of the traits studied (Sday, Gain40, EW40, and EN40) in the last generational stage was moderate to high, ranging from 0.23 to 0.74. Genetic and phenotypic correlations between the age of first egg (Sday) and the number of eggs laid up to 40 weeks of age (EN40) were negative, which could be beneficial to the production system in selecting for early sexual maturity and improving the egg production of indigenous chickens.

## Figures and Tables

**Table 1:** Characteristics of data on laying traits of Taiwan indigenous chicken lines.

Line	No of Laid Hen Recorded	Paternal Lineage			Maternal Lineage		
		Sire	PGS	PGD	Dam	MGS	MGD
L7	2390	190	116	166	678	219	424
L9	2417	186	116	157	735	213	459
L11	2982	204	136	174	826	270	540
L12	2045	182	113	148	651	221	406

PGS: paternal grand-sire, PGD: paternal grand-dam, MGS: maternal grand-sire, MGD: maternal grand-dam.

**Table 2:** Descriptive statistics for laying-related traits of Taiwan indigenous chicken lines.

Line	Trait	N	Mean	Min	Max	Quantile		CV	Skewness	Kurtosis
						0.05	0.95	(%)		
L7	W16, gm	2265	1627.4	580	2630	1112	2198	20.9	0.13	−0.65
	Sday, day	2390	148.5	116	209	131	169	7.9	0.71	1.38
	Gain40, gm	2379	439.0	−857	1747	−33	1005	74.8	0.31	−0.23
	EW40, gm	2169	45.5	27	66	37.6	53.2	10.7	0.02	0.20
	EN40, egg	2389	86.9	3	133	50	114	22.6	−0.75	0.85
L9	W16, gm	2312	1493.7	815	2324	1093	1866	15.7	−0.07	−0.13
	Sday, day	2418	143.4	116	210	126	164	8.1	0.56	0.74
	Gain40, gm	2413	397.7	−870	1897	−122	1017	86.0	0.37	0.42
	EW40, gm	2139	46.7	26	76	40.6	53.0	8.4	−0.07	2.60
	EN40, egg	2413	83.3	1	143	38	114	27.5	−0.87	0.87
L11	W16, gm	2815	1564.7	824	2382	1136	1973	16.5	−0.03	−0.49
	Sday, day	2982	143.5	116	222	127	168	9.4	1.50	4.07
	Gain40, gm	2980	303.3	−702	1545	−62.5	748.5	84.9	0.63	1.27
	EW40, gm	2700	47.8	26	69	41.8	53.8	8.0	0.12	1.71
	EN40, egg	2979	88.2	1	145	41	119	26.8	−0.95	0.96
L12	W16, gm	1942	1616.4	650	2785	1095	2148	20.4	0.06	−0.66
	Sday, day	2045	151.1	121	211	130	180	10.2	0.75	0.42
	Gain40, gm	2043	234.3	−714	1599	−147	725	113.8	0.45	0.88
	EW40, gm	1829	47.3	32	72	40.5	54.2	8.8	0.12	1.09
	EN40, egg	2044	77.1	2	132	34	106	28.0	−0.90	0.89

Note: W16: body weight at l6 weeks of age, Sday: age at first egg, Gain40: body weight changes between Sday and 40 weeks of age, EW40: mean egg weight at 40 weeks of age, EN40: egg number up to 40 weeks of age.

**Table 3:** Inbreeding coefficient summary of Taiwan indigenous chicken by line and generational stage.

Generation Stage	Generation	L7		L9		L11		L12		*p*-Value
		Mean	Range	Mean	Range	Mean	Range	Mean	Range	
GS1	G1	0	-	0	-	0	-	0	-	-
GS2	G2–G4	0.07	0–0.31	0.09	0–0.38	0.10	0–0.38	0.09	0–0.34	0.043
GS3	G5–G7	0.09	0.02–0.15	0.08	0.02–0.15	0.11	0–0.26	0.13	0–0.28	<0.0001
GS4	G8–G10	0.18	0.11–0.34	0.15	0.10–0.33	0.19	0.11–0.37	0.21	0.16–0.37	<0.0001
GS5	G11–G13	0.25	0.17–0.41	0.21	0.14–0.35	0.24	0.19–0.34	0.26	0.20–0.43	<0.0001
GS6	G14–G16	0.28	0.23–0.49	0.27	0.22–0.43	0.29	0.24–0.49	0.31	0.27–0.48	<0.0001
GS7	G17–G18	0.33	0.28–0.52	0.28	0.24–0.44	0.31	0.28–0.47	0.34	0.30–0.50	<0.0001

The *p*-value is the significance level of F-test obtained from mean inbreeding coefficient comparison among lines within generational stage.

**Table 4:** Estimates of heritability and SE of laying traits in Taiwan indigenous chicken lines by generational stage using multivariate animal model.

Line	Trait	Generational Stage						
		GS1	GS2	GS3	GS4	GS5	GS6	GS7
L7	Sday	0.31 (0.11)	0.61 (0.13)	0.25 (0.11)	0.44 (0.12)	0.20 (0.10)	0.46 (0.10)	0.28 (0.15) ^NS^
	Gain40	0.35 (0.07)	0.44 (0.14)	0.47 (0.12)	0.41 (0.10)	0.34 (0.09)	0.42 (0.07)	0.51 (0.12)
	EW40	0.54 (0.10)	0.75 (0.14)	0.74 (0.08)	0.80 (0.11)	0.56 (0.05)	0.42 (0.07)	0.32 (0.07)
	EN40	0.35 (0.10)	0.38 (0.14)	0.19 (0.10) ^NS^	0.20 (0.09)	0.15 (0.09) ^NS^	0.58 (0.05)	0.27 (0.16) ^NS^
L9	Sday	0.38 (0.13)	0.09 (0.08) ^NS^	0.17 (0.09) ^NS^	0.10 (0.13) ^NS^	0.29 (0.13)	0.42 (0.12)	0.23 (0.12) ^NS^
	Gain40	0.38 (0.14)	0.29 (0.09)	0.23 (0.09)	0.34 (0.14)	0.70 (0.12)	0.66 (0.06)	0.60 (0.07)
	EW40	0.42 (0.13)	0.38 (0.10)	0.44 (0.09)	0.57 (0.14)	0.60 (0.12)	0.51 (0.05)	0.74 (0.10)
	EN40	0.48 (0.12)	0.15 (0.07)	0.25 (0.10)	0.27 (0.14) ^NS^	0.52 (0.06)	0.12 (0.06)	0.00 (0.01) ^NS^
L11	Sday	0.36 (0.10)	0.24 (0.14)	0.38 (0.12)	0.32 (0.13)	0.22 (0.11)	0.28 (0.09)	0.37 (0.15)
	Gain40	0.31 (0.10)	0.17 (0.07)	0.57 (0.07)	0.52 (0.07)	0.45 (0.12)	0.36 (0.05)	0.30 (0.10)
	EW40	0.63 (0.11)	0.54 (0.08)	0.61 (0.09)	0.53 (0.04)	0.57 (0.07)	0.63 (0.07)	0.65 (0.11)
	EN40	0.49 (0.12)	0.31 (0.11)	0.17 (0.08)	0.09 (0.06) ^NS^	0.13 (0.06)	0.10 (0.06) ^NS^	0.52 (0.08)
L12	Sday	0.49 (0.11)	0.27 (0.10)	0.36 (0.11)	0.48 (0.14)	0.27 (0.14) ^NS^	0.32 (0.11)	0.28 (0.14)
	Gain40	0.34 (0.09)	0.15 (0.08) ^NS^	0.39 (0.10)	0.03 (0.05) ^NS^	0.47 (0.12)	0.48 (0.15)	0.34 (0.11)
	EW40	0.69 (0.09)	0.32 (0.06)	0.52 (0.15)	0.57 (0.11)	0.64 (0.09)	0.83 (0.08)	0.48 (0.14)
	EN40	0.65 (0.09)	0.25 (0.09)	0.18 (0.09)	0.19 (0.10) ^NS^	0.44 (0.15)	0.39 (0.18)	0.16 (0.10) ^NS^

Values in the parentheses are the standard error of heritability estimate. ^NS^: Heritability estimate is not significantly different from zero at the 5% level of significance.

**Table 5:** Genetic and phenotypic correlation estimates at generational stage by line in Taiwan indigenous chicken using multivariate animal model.

Line	Trait	Generational Stage							
		GS1 (G1)				GS7 (G17–G18)			
		Sday	Gain40	EW40	EN40	Sday	Gain40	EW40	EN40
L7	Sday	-	−0.54 *	−0.09	−0.27	-	−0.09	−0.38 *	−0.81 *
	Gain40	−0.18 *	-	0.07	0.23	−0.19 *	-	0.45	0.20
	EW40	−0.09	−0.03	-	−0.47 *	−0.13 *	0.13 *	-	−0.11
	EN40	−0.33 *	0.07	−0.03	-	−0.27 *	−0.03	0.04	-
L9	Sday	-	−0.33 *	−0.22	−0.46 *	-	0.31	0.52	−0.31
	Gain40	−0.26 *	-	0.30	0.72 *	−0.07	-	0.49 *	−0.30
	EW40	−0.14	0.07	-	−0.22	0.18 *	0.20 *	-	−0.96
	EN40	−0.27 *	0.36 *	0.13	-	−0.19 *	−0.11	0.02	-
L11	Sday	-	−0.12	−0.31	−0.72 *	-	−0.22	0.02	−0.70 *
	Gain40	−0.28 *	-	0.34 *	0.30	−0.09	-	−0.05	0.20
	EW40	−0.19 *	0.20 *	-	0.18	−0.03	−0.01	-	−0.24 *
	EN40	−0.35 *	0.04	0.17 *	-	−0.24 *	−0.12 *	−0.11	-
L12	Sday	-	−0.19	−0.26	−0.57 *	-	0.86 *	−0.50	−0.81 *
	Gain40	−0.19 *	-	0.01	0.13	0.08	-	−0.42	−0.40
	EW40	−0.11	−0.02	-	0.30	−0.09	−0.09	-	0.42
	EN40	−0.46 *	0.17 *	0.12 *	-	−0.29 *	−0.17 *	0.18 *	-

Note: Genetic and phenotypic correlations (*r_g_* and *r_p_*) within line are above and below the diagonal, respectively. SEs of *r_g_* and *r_p_* used for significance test were described by Groeneveld et al. and are (1 − *r_p_*^2^)/(N − 3)^1/2^, respectively. *: Estimate is significantly different from zero at the 5% level of significance.

## Data Availability

Data are available only upon agreement with Taiwan Livestock Research Institute and should be requested directly from the authors.
